# Complete Sequence and Analysis of Plastid Genomes of Two Economically Important Red Algae: *Pyropia haitanensis* and *Pyropia yezoensis*

**DOI:** 10.1371/journal.pone.0065902

**Published:** 2013-05-29

**Authors:** Li Wang, Yunxiang Mao, Fanna Kong, Guiyang Li, Fei Ma, Baolong Zhang, Peipei Sun, Guiqi Bi, Fangfang Zhang, Hongfan Xue, Min Cao

**Affiliations:** 1 Key Laboratory of Marine Genetics and Breeding, College of Marine Life Sciences, Ocean University of China, Qingdao, China; 2 Key Laboratory for Sustainable Utilization of Marine Fisheries Resources, Ministry of Agriculture, Yellow Sea Fisheries Research Institute, Chinese Academy of Fishery Sciences, Qingdao, China; Nanjing Forestry University, China

## Abstract

**Background:**

*Pyropia haitanensis* and *P. yezoensis* are two economically important marine crops that are also considered to be research models to study the physiological ecology of intertidal seaweed communities, evolutionary biology of plastids, and the origins of sexual reproduction. This plastid genome information will facilitate study of breeding, population genetics and phylogenetics.

**Principal Findings:**

We have fully sequenced using next-generation sequencing the circular plastid genomes of *P. hatanensis* (195,597 bp) and *P. yezoensis* (191,975 bp), the largest of all the plastid genomes of the red lineage sequenced to date. Organization and gene contents of the two plastids were similar, with 211–213 protein-coding genes (including 29–31 unknown-function ORFs), 37 tRNA genes, and 6 ribosomal RNA genes, suggesting a largest coding capacity in the red lineage. In each genome, 14 protein genes overlapped and no interrupted genes were found, indicating a high degree of genomic condensation. *Pyropia* maintain an ancient gene content and conserved gene clusters in their plastid genomes, containing nearly complete repertoires of the plastid genes known in photosynthetic eukaryotes. Similarity analysis based on the whole plastid genome sequences showed the distance between *P*. *haitanensis* and *P*. *yezoensis* (0.146) was much smaller than that of *Porphyra purpurea* and *P*. *haitanensis* (0.250), and *P*. *yezoensis* (0.251); this supports re-grouping the two species in a resurrected genus *Pyropia* while maintaining *P. purpurea* in genus *Porphyra*. Phylogenetic analysis supports a sister relationship between Bangiophyceae and Florideophyceae, though precise phylogenetic relationships between multicellular red alage and chromists were not fully resolved.

**Conclusions:**

These results indicate that *Pyropia* have compact plastid genomes. Large coding capacity and long intergenic regions contribute to the size of the largest plastid genomes reported for the red lineage. Possessing the largest coding capacity and ancient gene content yet found reveal that *Pyropia* are more primitive multicellular red algae.

## Introduction

Plastids are the photosynthetic organelles that provide essential energy for algae, land plants, and some protozoa. In addition to photosynthesis, several other metabolic pathways are present within plastids, including the biosynthesis of fatty acids, amino acids, pigments, and vitamins [1]. Plastids possess their own genetic systems and their own genomes [1]. The origin and evolution of plastid genomes, or plastids themselves, have long occupied significant areas within the biological sciences. It is now generally accepted that plastids originated through a single primary endosymbiotic event, whereby a free-living prokaryote engulfed and retained a foreign cyanobacterium inside a vacuole [2], [3]. The highly conservative nature and slow evolutionary rate of the plastid genome demonstrated that the genome was uniform enough to perform comparative studies across different taxa, but sufficiently divergent so as to capture evolutionary events; thus making it a suitable and invaluable tool for molecular phylogeny and molecular ecology studies [4].

*Pyropia haitanensis* Chang et Zheng and *P. yezoensis* Ueda are two economically important marine crops grown in harsh intertidal habitats. For hundreds of years, they have been cultivated in east Asian countries, such as China, Korea and Japan, where they today constitute one of the largest aquaculture industries [5]. Developing new commercial strains with competitive advantageous traits (including stress-tolerance and disease-resistance), continues to be a pressing need. So far, the breeding of *Pyropia* by traditional selection and hybridization methods has progressed such that strains showing high growth rates and economically valuable characteristics have been obtained, but these methods have limitations in terms of longer breeding cycles and the isolation of strains carrying heat-stress tolerance or disease resistance [6]. Molecular breeding techniques based on genomic and genetic information are expected to create new breeds of cultivars with a better flavor of the dried nori, higher tolerance to stress, higher resistance to disease, and a deeper color even under the low-nutrient conditions in *Pyropia* farms.

*Pyropia* species are also considered to be research models for studying physiological ecology of intertidal seaweed communities, evolutionary biology of plastids, diversity of reproduction, and origins of sexual reproduction [7]. Extensive research via sequencing of ESTs [8]–[10] and establishment of a transcriptomic database [11], [12] was recently performed in *Pyropia* and *Porphyra* to elucidate various mechanisms of biological importance to the two genera. In addition, research has also increased the availability of genetic resources for *Pyropia*, such as the construction of a genetic linkage map [13], and genetic analysis of artificial pigmentation mutants [14], [15]. However, systematic molecular investigations and genomic information on *Pyropia* remain incomplete.

There is insufficient sequence data available for the red algae, in terms of both nuclear and plastid genome sequences. In public databases, more than 310 complete plastid genomes are available from land plants and green algae, whereas fewer than 35 sequences belong to red algae or photosynthetic chromalveolate species. Only five complete plastid sequences have been reported for red algal species, including representatives of Cyanidiophyceae (*Cyanidium caldarium* and *Cyanidioschyzon merolae*) [16], [17], Bangiophyceae (*Porphyra purpurea* and *P*. *yezoensis*) [18, [NC_007932]], and Florideophyceae (*Gracilaria tenuistipitata* var. liui) [19]. The information of plastid genomes for the order Bangiales is very limited and represented only by the complete sequence from *P*. *purpurea* and *P*. *yezoensis*.

Information on the complete plastid genome sequence is not only important for evolutionary studies but also for crop improvement. In higher plants, several important agronomic traits are found to be associated with the plastid genome, which is usually inherited uniparentally [20], [21]. Chloroplast genetic engineering has also been achieved successfully in some important crops such as carrot, rice, and tobacco [22]–[24]. However, the lack of a stable transformation system has hampered elucidation of gene function and molecular breeding of *Pyropia*; and the availability of plastid genome sequences should be useful in developing genetic engineering, including chloroplast transformation [25], [26]. Moreover, characterization of plastid genome polymorphisms is commonly used for phylogeographic, population genetic and forensic analyses in plants [27]–[29]. In general, plastid genomes have provided an efficient platform not only for the improvement of agronomic traits but also for the conservation of the population genetic diversity. Therefore, additional plastid genomes from novel taxa will not only advance our understanding of the diversity of the order Bangiales and the evolution of the red algae, but will also open up the possibility for genetic engineering of economically important red seaweed.

Here we report the complete plastid genome sequence of *P*. *haitanensis*, and *P*. *yezoensis*, with analysis of their genome structure and gene content. These sequences represent the fully characterized plastid genomes from the newly described genus *Pyropia*. In addition, a comparative analysis of the plastid genomes of the two species and *P*. *purpurea*, and plastid phylogenies based on the genomic data currently available for algae and higher plants, were conducted to reveal taxonomic relationships of the Bangiales and evolution of the red algal plastid genomes. Also, we herein demonstrate the conservative properties and variability of plastid genomes among the red and green lineages.

## Materials and Methods

### Algal materials and DNA extraction

Unialgal conchocelis colonies derived from a single conchocelis filament (sporophytes) of *P*. *haitanensis* (strain PH-38) and *P*. *yezoensis* (strain RZ-58) were cultivated at 24°C and 20°C, respectively, under fluorescent lights (12 hr light:12 hr dark; 20 µmol photons m^−2^s^−1^) and constant aeration in sterilized filtered seawater that was changed weekly and supplemented with Provasoli's enrichment [30]. The sporophytes obtained were washed with filtered seawater three times and then wiped off with filter paper before use. Total DNA from the two red algal species was prepared from filamentous sporophytes using a Plant Genomic DNA Kit (Tiangen, China) following the manufacturer's protocol.

### Genome sequencing, assembly, annotation and analysis

About five µg total DNA from sporophytes of the two red algae were randomly sheared into fragments for sequencing. For each species, one half of a standard 454 sequencing run was performed on a Roche GS-FLX sequencer. The adaptors, low-complexity sequences, sequences with an excess of errors (more than 4% of N) and very short sequences (less than 100 bp) were trimmed by using the LUCY [31] and Seqclean (http://compbio.dfci.harvard.edu/tgi/). The duplicated reads were eliminated by using CD-HIT-454 software [32]. The pre-processed sequences were then subject to assembling using the program Newbler v2.5.3 (Roche 454 Life Sciences, Branford, CT) with default assembly parameters. The estimation of misassembled sequences due to repetitive regions in the genomes was performed using the integrated pipeline amosvalidate [33]. Then all the correctly assembled sequences were aligned to the *P*. *yezoensis* (GenBank Accession No. NC_007932.1) and *P*. *purpurea* (GenBank Accession No. NC_000925.1) plastid genomes (used as reference genomes) using the BLAST program (http://blast.ncbi.nlm.nih.gov/), and aligned contigs (query coverage≥60% and E-value≤1e-10) were ordered according to the reference genomes. These plastid contigs were extracted from the rest of the sequence data, and the gaps between the contigs in each plastid genome were filled by PCR and Sanger sequencing using the primers mentioned in Table S1. The sequences of the circular plastid genomes were completed by manual assembly.

Protein coding genes and putative open reading frames (ORFs) were annotated using NCBI ORF-finder (http://www.ncbi.nlm.nih.gov/gorf/gorf.html) and BLASTX and BLASTN searches at NCBI (http://www.ncbi.nlm.nih.gov/). Ribosomal RNA genes were identified by database comparison (BlastN), and tRNA genes were identified using tRNAScan-SE [34]. The circular genome map was constructed using OGDRAW (http://ogdraw.mpimp-golm.mpg.de/cgi-bin/ogdraw.pl). To verify the assembly and annotation, the randomly selected sequence regions (including homopolymer regions and coding gene-containing regions) in each plastid genome were confirmed by PCR and nucleotide sequencing using primers as mentioned in Table S1. The *P*. *haitanensis* and *P*. *yezoensis* sequencing reads were deposited in the NCBI Sequence Read Archive (SRA) database (http://www.ncbi.nlm.nih.gov/sra) under the BioProject accession numbers SRP020640 (experimental accessions SRX262853) and SRP020257 (experiment accessions SRX257139), respectively. The complete *P*. *haitanensis* and *P*. *yezoensis* plastid genomes are available under the following GenBank accession numbers: *P*. *haitanensis* (KC464603) and *P*. *yezoensis* (KC517072).

Sequence similarity of plastid genomes among *P*. *haitanensis*, *P*. *yezoensis,* and *P*. *purpurea* was measured using EMBOSS Stretcher (http://www.ebi.ac.uk/Tools/psa/emboss_stretcher/nucleotide.html) with the default settings. The plastid genome sequences of *P*. *haitanensis*, *P*. *yezoensis,* and *P*. *purpurea* were aligned using the multiple sequence alignment tools in CLUSTAL X version 1.81 [35] with the default settings to detect polymorphisms among these species. After alignment and gap removal, genetic distances were estimated by plastid genome sequences and coding sequences, respectively, among three species of the Bangiales under the Kimura 2-parameter model [36] using MEGA 5.0 [37]. Comparison of the plastid genomes among *C*. *merolae*, *P*. *haitanensis* and *Saccharina japonica* was made using the progressive Mauve algorithm using the default settings from MAUVE 2.3.1 software [38].

### Phylogenetic analyses

Phylogenetic analysis was conducted using 44 plastid protein sequences (*atpA*, *atpB*, *atpE*, *atpF*, *atpH*, *petA*, *petB*, *petD*, *petG*, *psaA*, *psaB*, *psaC*, *psaJ*, *psbA*, *psbB*, *psbC*, *psbD*, *psbE*, *psbF*, *psbH*, *psbI*, *psbJ*, *psbK*, *psbL*, *psbN*, *psbT*, *rpl14*, *rpl16*, *rpl2*, *rpl20*, *rpoA*, *rpoB*, *rps11*, *rps12*, *rps14*, *rps18*, *rps19*, *rps2*, *rps3*, *rps4*, *rps7*, *rps8*, *ycf3*, *ycf4*) from two cyanobacteria and 23 plastid genomes (Table S2). The concatenated protein sequences were aligned using the multiple sequence alignment tools in CLUSTAL X version 1.81 with the default settings, and sites containing gaps were removed. A Maximum Likelihood (ML) method was used to reconstruct phylogenetic trees using PHYML 3.0 [39] under cpREV [40] amino acid substitution matrices with 4 gamma-distributed rate categories. The neighbor-joining (NJ) approach was performed with the JTT [41] amino acid substitution matrix using MEGA 5.0. For both the ML and NJ methods, bootstrap support for each node was calculated using 1000 replicates. Finally, Bayesian analyses were performed with MrBayes3.2 [42] using the cpREV model. The Metropolis coupled Markov chain Monte Carlo algorithm from a random starting tree was initiated in the Bayesian inference and run for 300,000 generations, discarding the first 25% as “burn-in”. Four chains were run simultaneously, of which three were heated and one was cold. A consensus phylogeny was made with the “post burn-in” trees for the protein data set to determine the posterior probabilities at the different nodes.

## Results

### Sequencing and genome assembly

Using the 454 sequencing technology, a total of 502,634 and 554,607 raw reads, with average read length of 371 bp and 378 bp, were analyzed to generate 186,334,425 bp and 209,761,219 bp of sequence in *P*. *haitanensis* and *P*. *yezoensis*, respectively. After quality filters, 382,608 high-quality reads in *P*. *haitanensis* were assembled resulting in 12,413 contigs and 164,841 singlets with average lengths of 1,571 bp (N50 = 1,828 bp) and 381 bp (N50 = 447 bp), respectively. And 409,726 high-quality reads in *P*. *yezoensis* were assembled into 9,549 contigs and 176,849 singlets with average lengths of 1,105 bp (N50 = 1,328 bp) and 380 bp (N50 = 447 bp), respectively. After screening the assembled sequences through the estimation of misassembled sequences and alignment with the reference genomes, a total of 17 contigs (the mean coverage of contigs was 70) corresponding to *P*. *haitanensis* plastid DNA had homology with the reference genomes with an average contig size of 11,026 bp (ranging from 819 to 45,008 bp); and 28 contigs (the mean coverage of contigs was 73) corresponding to *P*. *yezoensis* plastid DNA had homology with the reference genomes with an average contig size of 6,803 bp (ranging from 1,175 to 21,855 bp). The gaps between these contigs in each plastid genome were filled via direct sequencing of PCR products, and then the complete plastid genomes of *P*. *haitanensis* and *P*. *yezoensis* were obtained.

To validate the assembly, 41 and 10 sequence regions (including homopolymer regions and coding gene-containing regions) in plastid genomes of *P*. *haitanensis* and *P*. *yezoensis*, respectively, were confirmed by PCR amplifications and Sanger sequencing. We compared these sequences directly to the assembled genomes, observing no nucleotide mismatches or indels. This result also validated the accuracy of our genome sequencing and assembly.

### Organization and gene content of the *P*. *haitanensis* and *P*. *yezoensis* plastid genomes

**Figure 1:**
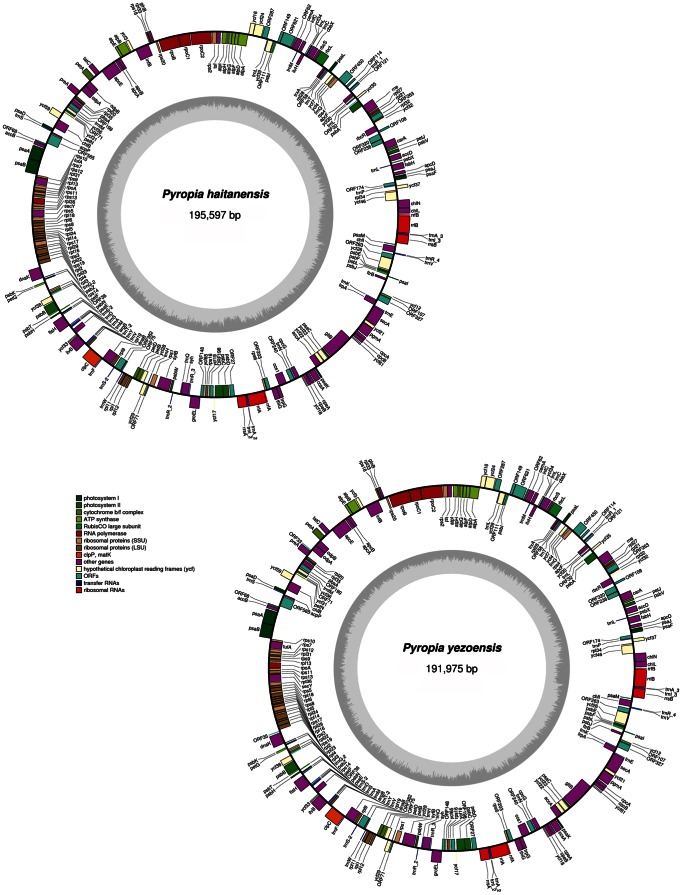
Plastid genome maps of *P. haitanensis* and *P. yezoensis*. Genes present on the inside of the circles are transcribed clockwise, whereas those on the outside counter clockwise. Annotated genes are colored according to the functional categories shown in the legend and the tRNA genes are indicated by the single-letter code of the corresponding amino-acid.

The plastid genomes of *P. haitanensis* and *P*. *yezoensis* were 195,597 and 191,975 base pairs (bp) in size, respectively. Figure 1 illustrates the gene maps of *P. haitanensis* and *P*. *yezoensis*. The size difference between the two genomes was primarily due to the presence of longer intergenic regions in *P. haitanensis*. The intergenic region accounted for about 16.5% of the entire genome, whereas only 14.9% of the *P*. *yezoensis* plastid DNA was intergenic. Overall, the GC content was 32.98% for *P. haitanensis* and 33.09% for *P*. *yezoensis*, which is comparable to that of *C*. *caldarium* (32.7%), *G*. *tenuistipitata* (29.1%), *P*. *purpurea* (33.0%) and *Guillardia theta* (33.0%).

The plastid genomes of *P. haitanensis* and *P*. *yezoensis* contained two small (approximately 4.8 kb) direct non-identical repeats that possessed two ribosomal operons encoding 16 S, 23 S and 5 S rRNAs. The two plastid genomes were predicted to encode a total of 211 and 213 protein-coding genes, respectively; and 37 tRNA genes, when all duplicated genes were counted. A total of these coding genes indicated a largest coding capacity in the red lineage when compared to that of other red algal and red-derived plastid genomes (i.e., *P*. *purpurea* (253 genes), *G*. *tenuistipitata* (237 genes), *C*. *merolae* (243 genes), *C*. *caldarium* (230 genes), *Ectocarpus siliculosus* (185 genes) and *Phaeodactylum tricornutum* (170 genes)). In addition, 29 and 31 open reading frames (ORFs) were identified with a threshold of 90 bp in *P. haitanensis* and *P*. *yezoensis*, respectively. The only difference in gene content between the two genomes was the absence of two additional ORFs (*ORF33*, *ORF35*) with unknown function in *P. haitanensis*, which encoded putative proteins of 33 and 35 amino acids. For the two plastid genomes, almost all of the genes were present in a single copy with the exception of two ORFs (*ORF71* and *ORF263*), and this gene content is typical for the Bangiales plastid genome. No interrupted genes were identified in either plastid genome. Overlaps involving *psbC*-*psbD*, *atpD*-*atpF*, *ycf24*-*ycf16*, *rps19*-*rpl2*, *rpl23*-*rpl4*, *carA*-*ORF238*, and *rpl24*-*rpl14* with one overlapping nucleotide are common to *P. haitanensis* and *P*. *yezoensis*. These results indicated the two genomes were the epitome of compactness (83.5% and 85.1% are coding sequence). This is similar to the situation in *Odontella sinensis* (84.3%), *G*. *theta* (90%), *G*. *tenuistipitata* (84.1%) and *C*. *caldarium* (88.4%), but contrasts with green plants such as rice (68%), *Sesamum indicum* (58%) and *Erycina pusilla* (53.27%).

The ochre termination codon TAA was used in *P. haitanensis* and *P*. *yezoensis* 75.8% and 78.9% of the time, with amber (TAG) used 17.5% and 16.4%, and opal (TGA) codons used 6.6% and 4.7%, respectively. In 11 cases a valine start codon (GTG) was used rather than methionine (*ORF320*, *rbcS*, *infC*, *rpoC2*, *accB*, *rpoA*, *rps13*, *rps3*, *rpl3*, *chll*, and *rps20*) for both plastid genomes, with a TTG start codon and ATA start codon used in five genes (*petJ*, *ORF263*, *ycf65*, *ORF27*, *trpG*) and *dnaB*, respectively.

### Comparison of *P. haitanensis*, *P. yezoensis* and *P. purpurea* plastid genomes

**Figure 2:**
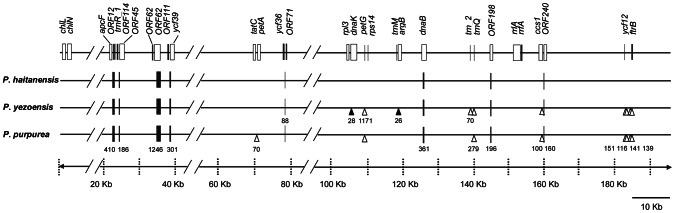
Comparison of plastid genomes of *P. haitanensis*, *P. yezoensis*, and *P. purpurea*. Insertions longer than 25 bp or deletions longer than 70 bp in *P*. *yezoensis* and *P*. *purpurea* in comparison with *P. haitanensis* are labeled as black triangles or white triangles individually. Highly diverse sequence regions larger than 80 bp are labeled with black blocks. Numbers indicate the longest length of comparative deletions, insertions, or diverse sequence regions of three Bangiales species.

To further clarify the taxonomy of *P*. *haitanensis*, *P*. *yezoensis* and *P*. *purpurea*, the genome structure, gene content and sequence similarity of the three plastid genomes were compared. The percentages of intergenic regions in *P. haitanensis* plastid genome were greater than those of *P*. *yezoensis* (14.9% of the genome) and *P*. *purpurea* (about 14.4% of the genome) (GenBank Accession No. NC_000925.1). Furthermore, most insertions, deletions or diverse sequence regions occurred within intergenic regions (Figure 2). For example, 26- and 28-bp insertions were found in the intergenic region of *trnM*-*argB* and *rpl3*-*dnaK* in *P*. *yezoensis*, respectively. Deletions longer than 70 bp in the *P*. *yezoensis* and *P*. *purpurea* sequences, compared to *P. haitanensis*, were found at 9 loci and represented about 2167 bp, or 1.11% of the *P*. *haitanensis* genome. Significantly, the areas of high diversity among the compared genomes were found in the region located within ORFs or intergenic regions between ORFs and other genes, which represented 1.51% of the *P*. *haitanensis* sequence. These results indicated that indels and diversity are common in intergenic regions of the Bangiales plastid genomes, and deletions mainly occur in *P*. *yezoensis* and *P*. *purpurea*. Additionally, Gene content of the three plastid genomes was similar, with 252 conserved functional genes within all three plastid genomes. However, four ORFs of unknown function (*ORF32*, *ORF33*, *ORF35*, and *ORF36*) were absent in *P*. *purpurea* plastid genome, and of which two ORFs (*ORF32*, *ORF36*) were shared by *P*. *haitanensis* and *P*. *yezoensis*. *P*. *purpurea* plastid also included an RNAse P RNA gene not present in the other genomes.

Although the genome structure and gene content among the three species were similar, sequence similarity was found to be divergent between the two *Pyropia* species and *P*. *purpurea*. The plastid genome of *P*. *haitanensis* shared 85.6% and 78.8% identity with *P*. *yezoensis* and *P*. *purpurea*, respectively. The genetic distance, revealed by the plastid genome sequence dataset, was smaller (0.146) between *P*. *haitanensis* and *P*. *yezoensis* (belonging to the same genus), compared to those from *P*. *haitanensis* and *P*. *purpurea* (0.250), *P*. *yezoensis* and *P*. *purpurea* (0.251); similarly, the genetic distance, revealed by the coding sequence of the plastid genomes, was smaller (0.138) between *P*. *haitanensis* and *P*. *yezoensis*, compared to those from *P*. *haitanensis* and *P*. *purpurea* (0.228), *P*. *yezoensis* and *P*. *purpurea* (0.230). With respect to overlaps in plastid genomes, an overlap of four bases between *carA* and *ORF238* was present in both *P. haitanensis* and *P*. *yezoensis*, but absent in *P*. *purpurea*. These results showed that *P*. *haitanensis* is closer to *P*. *yezoensis* than *P*. *purpurea* in the Bangiales.

### The conservative property and variability of plastid genomes among red and green lineages

Compared with the previously published red and green photosynthetic plastid genomes (with the exception of the dinoflagellates [43]) (Table S3), a common core set of 40 genes was found to be shared by all the genomes. These genes mainly encode essential plastid proteins involved in transcription, translation and photosynthetic metabolism, such as components of photosystem I and II complexes, ATP synthase and the cytochrome complex. Meanwhile, 30 additional protein-coding genes were also found in many plastid genomes of both red and green lineages. Of the 85 genes mainly encoded by plastid genomes of red lineage, 22 genes were present in all plastid genomes of red lineage, but all were absent from the green lineage chloroplast DNAs (cpDNAs). Most of the missing genes from green lineage cpDNAs (many of which transfer from the chloroplast to nucleus; i.e., *atpD*, *psaF*, *dnaK*, *groEL*, *rbcS*, *secY*, *infB* and *infC*), are involved in DNA replication (*dnaK* and *dnaB*) and cell/organelle division (*ftsH* and *groEL*), transcription and translation (i.e., *rpl6*, *rpl21*, *infB*, *infC*), photosynthetic metabolism (i.e., *psaF*, *psbV*, *psaE* and *psbX*), and unidentified functions (ycf genes) (Table S3). Interestingly, the 26 genes, mainly involved in components of phycobilisomes (i.e., *cpeA*, *apcA*, *B*, *D*, *E* and *F*, *cpcA*, *B* and *G*) and biosynthesis of amino acids (i.e., *trpA* and *G*, *argB*, *gltB*) and fatty acids (i.e., *accA*, *B*, *D*), were only present in red algae, whereas they were absent from the plastid genomes of chromists and green lineage (with the exception of *accD* found in *Bryopsis hypnoides* and *Oryza rufipogon*) (Table S3). Within red algae, the gene *glnB* was found to be absent from the *Gracilaria* plastid genome, whereas it was present in unicellular red algae, the Bangiales and two cyanobacteria (*Synechocystis* sp. PCC 6803 and *Prochlorococcus marinus*). Additionally, of all of the 31 unique ORFs with unknown function encoded by the *Pyropia* plastid genomes, four (*ORF32*, *ORF33*, *ORF35* and *ORF36*) were not found in *Porphyra* plastid genomes. These ORFs shared low similarity to the cyanobacterial sequences or no similarity to the sequences in public database. Thus, the *Pyropia* maintain an ancient gene content in their plastid genomes, containing nearly the complete repertoires of plastid genes known in photosynthetic eukaryotes.

**Figure 3:**
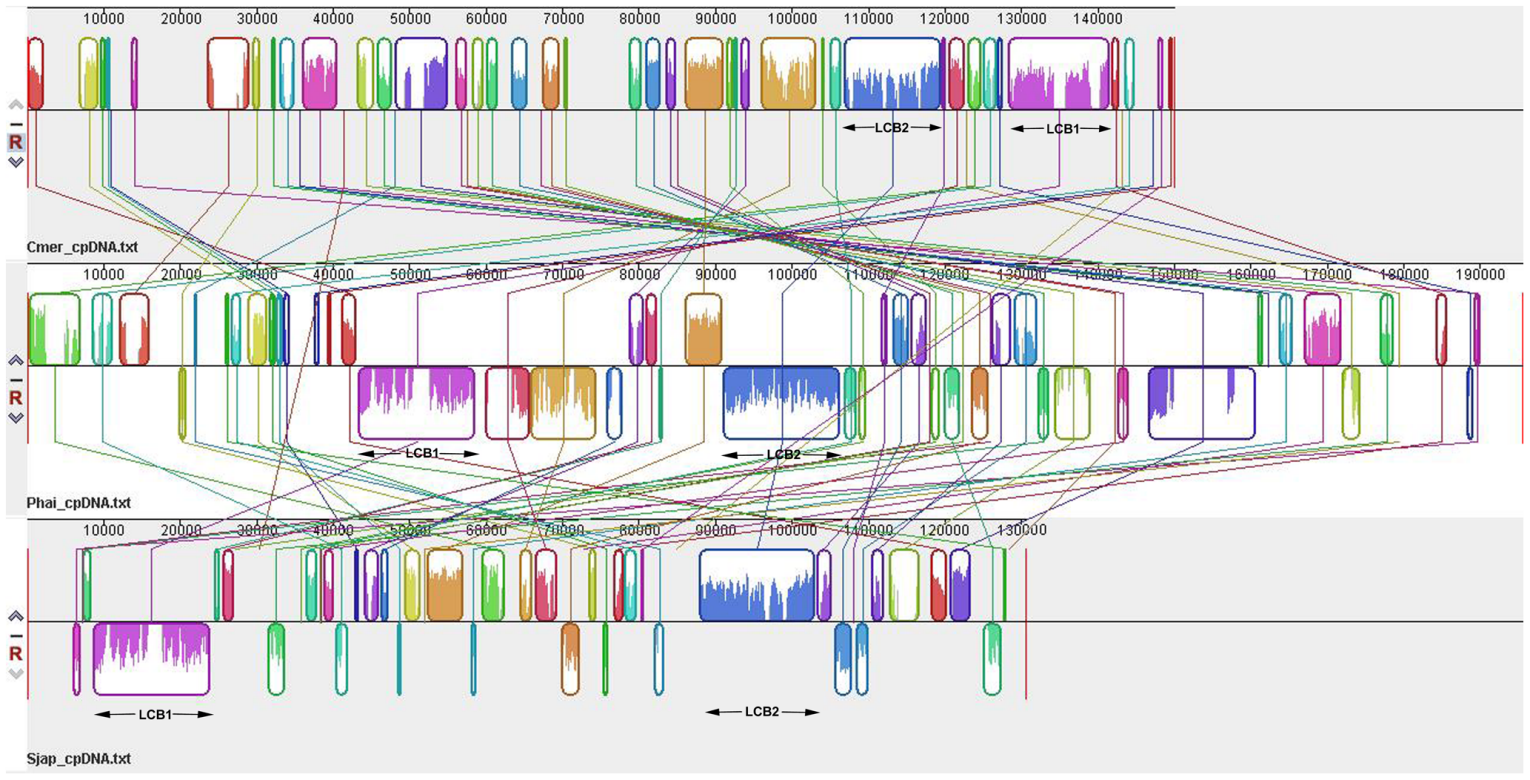
MAUVE genome comparison among *C merolae* (upper), *P. haitanensis* (middle), and *S. japonica* (lower) plastid genomes. Locally collinear blocks (LCBs) identified by MAUVE are color-coded; links between LCBs are indicated by the thin colored lines. In the middle and lower portion of the Figure, *P*. *haitanensis* and *S*. *japonica* LCBs lying below the black line have an inverse orientation relative to *C*. *merolae*.

With respect to gene organization, many gene clusters were shared specifically among most of the red photosynthetic plastid genomes. For example, two large, local collinear blocks (LCBs) were conserved with respect to both gene identity and order among *C*. *merolae*, *P*. *haitanensis* and *Saccharina japonica* plastid genomes (Figure 3). One block extended between *rpoB* and *atpA* and covered 7.9% of the plastid genomes of *P*. *haitanensis*. The second block contained a large proportion of ribosomal protein-coding genes, and covered up to 7.9% of the plastid genomes of *P*. *haitanensis*. Other gene clusters were also shared by many red algae and chromists (i.e., *psbE*-*psbF*-*psbL*-*psbJ* and *apcE*-*apcA*-*apcB*); as were gene pairs (i.e., *psaA*-*psaB*, *psbD*-*psbC*, *atpB*-*atpE*, *rbcL*-*rbcS*, *petB*-*petD*, *cpcB*-*cpcA*, *cpeB*-*cpeA* [not in Cyanidiales], and *odpA*-*odpB*), as well as one ancestral gene pair (*psbB*-*psbT*). In addition, the gene pairs *cpcB*-*cpcA*, *cpeB*-*cpeA* and *apcE*-*apcA*-*apcB* clusters were missing from plastid genomes of chromists. Many of these clusters, such as *rpoB*-*rpoC1*-*rpoC2*-*rps2*-*tsf*-*atpI*-*atpH*-*atpG*-*atpF*-*atpD*-*atpA*, *rbcL*-*rbcS*, *apcE*-*apcA*-*apcB*, *cpcB*-*cpcA*, *cpeB*-*cpeA*, and *odpA*-*odpB*, we believe to have been completely lost or reduced due to gene transfer to the nucleus in green lineage.

### Phylogeny of *P. haitanensis* and *P. yezoensis* based upon plastid genomes

**Figure 4:**
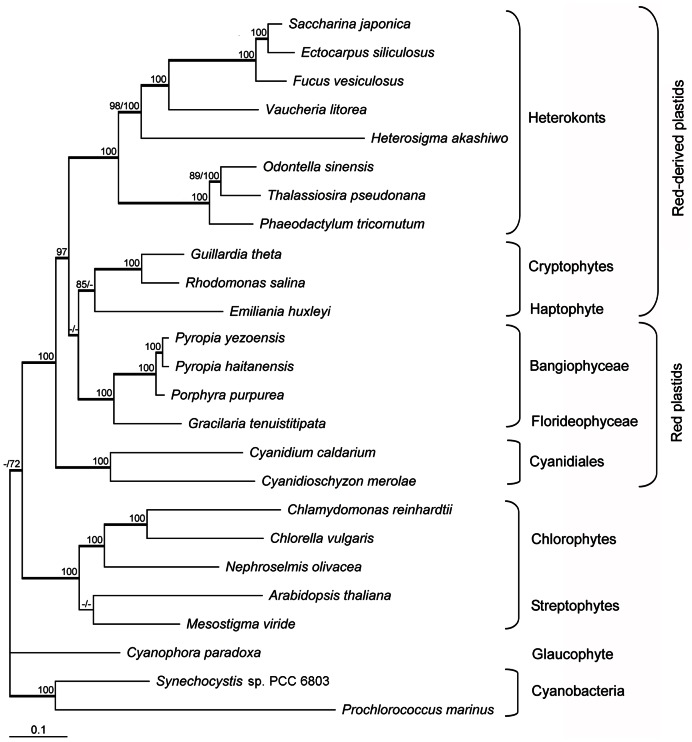
Maximum likelihood tree based on 44 concatenated proteins from 25 complete plastid or cyanobacterial genomes. ML and Neighbor Joining trees were constructed based on 8,628 amino-acid sites using cpREV and JTT matrices, respectively. When above 65% and different, bootstrap values (1000 replicates) are provided for ML (first value) and NJ (second value) analyses. The thick branches represent ≥0.9 posterior probability for Bayesian inference analysis.

In order to elucidate the overall position of *P*. *haitanensis* and *P*. *yezoensis* plastids in the phylogeny of algae/land plants, a global analysis was performed with a subset of 25 taxa and 8,628 amino-acid positions. Twenty-five organisms were included as representatives of algae and higher plants, containing cyanobacteria, the green lineage (streptophyte and chlorophyte lineage), the red lineage (red and chromist lineage), as well as glaucophyte *Cyanophora* (Table S2). All but three of the nodes in the trees were well resolved and supported by all three phylogenetic methods (Figure 4). Three distinct lineages were identified: the red lineage, the green lineage, and the Glaucophyte. Among plastids of red lineage, the clade uniting *P*. *haitanensis* and *P*. *yezoensis* received strong bootstrap support, and emerged as a sister group to *Porphyra* (*P*. *purpurea*) with high confidence using all methods; as did the Florideophyceae and Bangiophyceae. Moreover, (Florideophyceae + Bangiophyceae) was clustered with (haptophyte + cryptophytes) with high confidence in Bayesian analysis, and low bootstrap support (59%), or without support in the ML and NJ trees, respectively. The clade grouping (Florideophyceae + Bangiophyceae) and (haptophyte + cryptophytes) was located close to a clade of all heterokont plastids with strong bootstrap support (97%). In addition, the branching order of heterokonts, haptophyte and cryptophytes showed different topologies in the ML, NJ and Bayesian analyses. In the NJ topology, the haptophyte *E*. *huxleyi* emerged as the closest branch to all heterokont algae with poor bootstrap support (52%); but in the ML and Bayesian trees, it formed a strongly supported clade with cryptophytes. In the other part of the tree, the Cyanidiales clustered together outside a well-supported clade that included the Bangiophyceae and Florideophyceae, together with the heterokonts, the cryptophytes and the haptophyte plastids.

In all our analyses, the Glaucophyte plastid from *Cyanophora* emerged at the base of the red plastids, red-derived plastids and green plastids. Among the green plastids examined in this study, *Mesostigma* and *Arabidopsis* were grouped together with low confidence in Bayesian analysis, and received very low bootstrap support or no support in the ML and NJ trees, respectively. Three completely sequenced members of Chlorophytes formed a strongly supported lineage, grouping with the Streptophytes.

## Discussion

### Characteristics of the *Pyropia* plastid genomes

The genome size differences and sequence divergence among the three Bagiales plastid genomes were mainly due to the nucleotide indels and variations in the intergenic regions. Many deletions led to smaller size of the *Porphyra* plastid genome compared with that of *Pyropia*. Several significantly longer intergenic regions were found between *petG*-*rps14*, *trnM*(CAU)-*argB*, trnR_2-trnQ, *ccs1*-*ORF240* and *ycf12*-*ftrB* in *Pyropia* plastid genomes by comparison to the *Porphyra* genome. In particular, *petG*-*rps14* and *ycf12*-*ftrB* were separated by 1288 bp and 1964 bp in *P*. *haitanensis*, respectively. The GC content of the two intergenic regions was 29.37% and 28.8%, which was lower than the average GC content (33.0%) of *Pyropia* plastid genomes. Interestingly, a similarity search of intergenic regions between *petG* and *rps14* against GenBank revealed that this fragment matched with a plasmid described from *Porphyra pulchra* [44]. As for the intergenic region between *ycf12* and *ftrB*, the first two hits were also two plasmids from *P*. *pulchra*. It seems that these two regions might have originated through plasmid integration, which may relate to the evolution of the *Pyropia* plastid genome. Additionally, a great number of unknown-function ORFs may contribute to the genomic divergence. These ORFs were found to share low similarity to the cyanobacterial sequences or no similarity to the sequences in public database. These imply that the ancient ORFs may have experienced minor evolutionary pressures, and accumulated a large number of mutations during the evolutionary history of the order Bagiales, and even be lost in other algae. The function of these ORFs, or whether they are pseudogenes in Bagiales plastid genomes, requires further study.

Among all the plastid genomes of the red lineage, *Pyropia* plastids have a significantly increased number of coding genes (protein-coding genes and RNA genes), indicating a largest coding capacity in the red lineage. Most of the genes found in red-derived plastid genomes have already been identified in *Pyropia* plastid genomes. Among the *Pyropia* genes that are not found on red-derived plastid genomes are genes involved in components of phycobilisomes and redox system, biosynthetic pathways, carbohydrate metabolism, cofactors and translation, and genes with unidentified function. Within the red algae, *Pyropia* plastid genomes also possess more protein-coding genes than do other red algae. Thus, the large coding capacity can, at least partially, explain the size of the largest plastid genomes of *Pyropia* in the red lineage because of a high proportion of coding gene-containing regions in *Pyropia* plastid genomes. The large protein-coding gene set in *Pyropia* may play a role in stabilizing the photosynthetic machinery against high stress related to adaption to the intertidal, and often harsh, environments. Furthermore, compared with *Gracilaria* [19], *Pyropia* maintain a surprisingly ancient gene content (i.e., *psbZ*, *glnB*, *chlB*, *chlL*, *chlN*) which is also present in cyanobacteria. The presence of both the largest coding capacity and ancient gene content reveals that *Pyropia* are more primitive multicellular red algae.

### Modification of genomic data from *P. yezoensis* plastid

In this study, the plastid genome of *P*. *yezoensis* was sequenced using next-generation sequencing technology and fully validated by PCR amplification combined with Sanger sequencing. Although the genome size of the *P*. *yezoensis* plastid reported here was only 23 bp longer than the genome data published in NCBI GenBank (NC_007932.1), a great number of significant differences were found not only in nucleotide variation but also in gene content between the two versions. For example, *ORF565*, *ORF68*, *ORF75* and *trnL* (32,757–32,841) were predicted in the genome reported here, whereas they were absent in the previously published version. The tRNA gene *trnL* (39,863–39,944) was predicted to be located between ycf39 and ORF287, whereas the previous version instead showed that the tRNA gene *trnT* was in this same region. Additionally, thirteen tRNA genes that were previously predicted to be located in these regions (*accD*-*psbV*, *ORF263*-*rpl21*, *ycf20*-*apcF*, *ycf7*-*psaL*, *infB*-*ycf3*, *apcE*-*tatC*, *rpl13*-*rpoA*, *psbZ*-*psbK*, *ycf38*-*psbB*, *trnD*-*trnS*, and *trnK*-*ORF327*), were not found this time. Comparing the two versions of the *P. yezoensis* plastid genome with those of *P. haitanensis* and *P*. *purpurea*; the number, type and location of the tRNA genes in our study were quite similar with their relative species (*P. haitanensis* and *P*. *purpurea*) instead of previously published version. The difference of gene content between the two versions might be due to different annotation tools or methods used in our study and earlier work, whereas the difference of DNA sequence might be owing to different strain/cultivar of *P. yezoensis* used.

### Plastid DNA replication in the red lineage

An intriguing finding in the plastid genomes of the red lineage was the presence of many genes involved in DNA replication and cell/organelle division, such as *dnaB* (a DNA helicase [17], [45]), *hlpA* (a chromatin-associated architectural protein [46], [47]), *ftsH* (a metalloprotease acting as a chaperone protein [48]), *minD* and *minE* (which prevent the creation of DNA-less “mini-cells” during division [45]), and *groEL* (a protein chaperone [49]). Also, the *dnaX* (a member of the hsp70 family) gene in the cryptophyte alga *Rhodomonas salina* plastid genome is believed to be the first instance of a putative DNA polymerase enzyme encoded in plastid DNA, and is acquired by lateral gene transfer [46]. It is recognized that most of these genes encoded in red lineage plastid genomes are rarely found in green lineage plastid genomes. Although plastid DNA polymerases have been purified and characterized enzymatically for higher plants [50], the process of plastid DNA replication is still very poorly understood. Interestingly, an unusually high proportion of plastid DNA was found in total cellular DNA isolated from *P*. *haitanensis* and *P*. *yezoensis* using the standard genomic DNA preparation protocol [51] in our laboratory (data not shown). Since only one or two large plastids can be observed in each of the cells during their life history, the only possible explanation here is that there existed a high copy number of genomes in the *Pyropia* plastids. Therefore, for many species of red algae, extracting large amounts of high-quality nuclear DNA may be a great challenge not only due to a significant presence of polysaccharides, but also due to the presence of large amounts of plastid DNA. The functions of large plastid genomes remain unknown; and the relationships between high proportion of plastid DNA or high copy number of plastid genomes and the plastid genes involved in DNA replication and cell/organelle division need further investigation.

### Evolution of red algal plastid genomes

It is generally appreciated that reconstructing the phylogenetic relationship of host cell lineages can be difficult, especially because large-scale horizontal gene transfers have occurred in some lineages during their evolutionary history [52]. Unlike nuclear genomes, however, large-scale horizontal gene transfers involving plastid genes appear to be exceedingly rare, with some exceptions [53]. Therefore, it is appropriate to reconstruct phylogenies based on the plastid-genome data set. Much previous research has been performed in understanding the phylogeny of Bangiales floras based on a single or small number of nuclear or plastid genes [54], [55]. Recently, a generic revision of the order Bangiales has been made based on molecular analyses using *rbc*L and *nr*SSU genes combined with worldwide taxon sampling [56]. In this study, the global plastid-based phylogeny analyses confirmed the new revision that separates the genus *Porphyra* into several genera and groups the two commercially valued species *P*. *haitanensis* and *P*. *yezoensis* to a resurrected genus *Pyropia* while maintaining *P. purpurea* in the genus *Porphyra*.

Although the monophyly of Bangiophyceae and Florideophyceae were again well supported in this study, in accordance with previous observations [57]–[59]; the branching order of (Florideophyceae + Bangiophyceae), (cryptophytes + haptophyte) and heterokonts remains unresolved. Our phylogenetic results do not support a preferential link between (Florideophyceae + Bangiophyceae) and (cryptophytes + haptophyte), either in terms of gene content or phylogenetic relationship. Moreover, the phylogenetic analyses do not strongly support a clade grouping (cryptophytes + haptophyte) and heterokont plastids, as reported in a previous study [60]. It is therefore difficult to state a precise phylogenetic relationship among the (Florideophyceae + Bangiophyceae), (cryptophytes + haptophyte), and heterokonts from this study. The uncertain phylogenetic results may be due to insufficient datasets and taxon sampling.

Additionally, Cyanidiales are an asexual, unicellular red algal class that thrives in acidic and high-temperature conditions in hot springs or near acidic sulfur fumes [61]. Cyanidiales are a well-established eukaryotic lineage that diverged earliest within the red algae approximately 1,370 Mya, prior to the red algal secondary endosymbiosis of chromists [62].Our phylogenetic analyses showed that the red algal ancestor of chromist plastids was more closely related to the more recently evolved red algae (Florideophyceae and Bangiophyceae) than to Cyanidiales, in agreement with the report by Sanchez-Puerta *et al.* [63].

In summary, the red algae constitute one of the most distinct eukaryotic groups, and will contribute more to our understanding of eukaryote evolution. Additional plastid genomes from red algae, but certainly also from other evolved red algae, will be required to fully resolve the hidden biodiversity among red algae and plastid phylogenies of the red lineage.

## Supporting Information

Table S1 Primers used for gap filling and assembly validation.
(DOC)

Table S2 Plastid and cyanobacterial genomes used in the gene content comparisons and the phylogenetic studies.
(DOC)

Table S3 Gene content comparisons between plastid genomes.
(DOC)

## References

[1] Martin W, Herrmann RG (1998) Gene transfer from organelles to the nucleus: how much, what happens, and why? Plant Physiol 118: 9–17. 9733521 10.1104/pp.118.1.9 PMC1539188

[2] Reyes-Prieto A, Weber APM, Bhattacharya D (2007) The origin and establishment of the plastid in algae and plants. Annu Rev Genet 41: 147–168. 17600460 10.1146/annurev.genet.41.110306.130134

[3] Bhattacharya D, Medlin L (2006) The phylogeny of plastids: a review based on comparisons of small-subunit ribosomal RNA coding regions. J Phycol 31: 489–498.

[4] Raubeson LA, Jansen RK (2005) Chloroplast genomes of plants. In: Henry RJ, Plant diversity and evolution: Genotypic and phenotypic variation in higher plants. Wallingford: CAB International. 45–68.

[5] Mumford TF, Miura A (1988) *Porphyra* as food: Cultivation and economics. In: Lemby CA, Walland JR, Algae and human Affairs. Cambridge: Cambridge University Press. 87–117.

[6] Mikami K, Hirata R, Takahashi M, Uji T, Saga N (2011) Transient transformation of red algal cells: breakthrough toward genetic transformation of marine crop *Porphyra* species. In: MarÃa Alvarez, Genetic Transformation. Croatia: InTech-Open Access Publisher. 241–258.

[7] Blouin NA, Brodie JA, Grossman AC, Xu P, Brawley SH (2011) *Porphyra*: a marine crop shaped by stress. Trends Plant Sci 16: 29–37. 21067966 10.1016/j.tplants.2010.10.004

[8] Nikaido I, Asamizu E, Nakajima M, Nakamura Y, Saga N, et al (2000) Generation of 10,154 expressed sequence tags from a leafy gametophyte of a marine red alga, *Porphyra* yezoensis. DNA Res 7: 223–227. 10907854 10.1093/dnares/7.3.223

[9] Asamizu E, Nakajima M, Kitade Y, Saga N, Nakamura Y, et al (2003) Comparison of RNA expression profiles between the two generations of *Porphyra yezoensis* (Rhodophyta), based on expressed sequence tag frequency analysis. J Phycol 39: 923–930.

[10] Fan XL, Fang YJ, Hu SN, Wang GC (2007) Generation and analysis of 5318 expressed sequence tags from the filamentous sporophyte of *Porphyra haitanensis* (Rhodophyta). J Phycol 43: 1287–1294.

[11] Yang H, Mao YX, Kong FN, Yang GP, Ma F, et al (2011) Profiling of the transcriptome of *Porphyra yezoensis* with Solexa sequencing technology. Chin Sci Bull 56: 2119–2130.

[12] Chan CX, Zäuner S, Wheeler G, Grossman AR, Prochnik SE, et al (2012) Analysis of *Porphyra* membrane transporters demonstrates gene transfer among photosynthetic eukaryotes and numerous sodium-coupled transport systems. Plant Physiol 158: 2001–2012. 22337920 10.1104/pp.112.193896 PMC3320202

[13] Xie CT, Chen CS, Xu Y, Ji DH (2010) Construction of a genetic linkage map for *Porphyra haitanensis* based on sequence-related amplified polymorphism and simple sequence repeat markers. J Phycol 46: 780–787.

[14] Yan XH, Aruga Y (2000) Genetic analysis of artificial pigmentation mutants in *Porphyra yezoensis* Ueda (Bangiales, Rhodophyta). Psychol Res 48: 177–187.

[15] Niwa K (2010) Genetic analysis of artificial green and red mutants of *Porphyra yezoensis* Ueda (Bangiales, Rhodophyta). Aquaculture 308: 6–12.

[16] Glöckner G, Rosenthal A, Valentin K (2000) The structure and gene repertoire of an ancient red algal plastid genome. J Mol Evol 51: 382–390. 11040290 10.1007/s002390010101

[17] Ohta N, Matsuzaki M, Misumi O, Miyagishima S, Nozaki H, et al (2003) Complete sequence and analysis of the plastid genome of the unicellular red alga *Cyanidioschyzon merolae* . DNA Res 10: 67–77. 12755171 10.1093/dnares/10.2.67

[18] Reith M, Munholland J (1995) Complete nucleotide sequence of the *Porphyra purpurea* chloroplast genome. Plant Mol Biol Rep 13: 333–335.

[19] Hagopian JC, Reis M, Kitajima JP, Bhattacharya D, de Oliveira MC (2004) Comparative analysis of the complete plastid genome sequence of the red alga *Gracilaria tenuistipitata* var. liui provides insights into the evolution of rhodoplasts and their relationship to other plastids. J Mol Evol 59: 464–477. 15638458 10.1007/s00239-004-2638-3

[20] Chung S-M, Gordon VS, Staub JE (2007) Sequencing cucumber (*Cucumis sativus* L.) chloroplast genomes identifies differences between chilling-tolerant and -susceptible cucumber lines. Genome 50: 215–225. 17546086 10.1139/g07-003

[21] Gordon VS, Staub JE (2011) Comparative analysis of chilling response in cucumber through plastidic and nuclear genetic effects component analysis. J Am Soc Hortic Sci 136: 256–264.

[22] Kumar S, Dhingra A, Daniell H (2004) Plastid-expressed betaine aldehyde dehydrogenase gene in carrot cultured cells, roots, and leaves confers enhanced salt tolerance. Plant Physiol 136: 2843–2854. 15347789 10.1104/pp.104.045187 PMC523346

[23] Kathuria H, Giri J, Nataraja KN, Murata N, Udayakumar M, et al (2009) Glycinebetaine-induced water-stress tolerance in *codA*-expressing transgenic indica rice is associated with up-regulation of several stress responsive genes. Plant Biotechnol J 7: 512–526. 19490479 10.1111/j.1467-7652.2009.00420.x

[24] Fouad W, Altpeter F (2009) Transplastomic expression of bacterial l-aspartate-α-decarboxy-lase enhances photosynthesis and biomass production in response to high temperature stress. Transgenic Res 18: 707–718. 19353301 10.1007/s11248-009-9258-z

[25] Verma D, Daniell H (2007) Chloroplast vector systems for biotechnology applications. Plant Physiol 145: 1129–1143. 18056863 10.1104/pp.107.106690 PMC2151729

[26] Daniell H, Kumar S, Dufourmantel N (2005) Breakthrough in chloroplast genetic engineering of agronomically important crops. Trends Biotechnol 23: 238–245. 15866001 10.1016/j.tibtech.2005.03.008 PMC3486632

[27] Besnard G, Khadari B, Baradat P, Bervillé A (2002) Combination of chloroplast and mitochondrial DNA polymorphisms to study cytoplasm genetic differentiation in the olive complex (*Olea europaea* L.). Theor Appl Genet 105: 139–144. 12582571 10.1007/s00122-002-0868-6

[28] Arroyo-García R, Ruiz-García L, Bolling L, Ocete R, López MA, et al (2006) Multiple origins of cultivated grapevine (*Vitis vinifera* L. ssp. *sativa*) based on chloroplast DNA polymorphisms. Mol Ecol 15: 3707–3714. 17032268 10.1111/j.1365-294X.2006.03049.x

[29] Guillaume B, Pilar H, Bouchaib K, Gabriel D, Vincent S (2011) Genomic profiling of plastid DNA variation in the Mediterranean olive tree. BMC Plant Biol 11: 80. 21569271 10.1186/1471-2229-11-80 PMC3115843

[30] West JA, McBride DL (1999) Long-term and diurnal carpospore discharge patterns in the Ceramiaceae, Rhodomelaceae, and Delessariaceae (Rhodophyta). Hydrobiologia 288/289: 101–113.

[31] Chou HH, Holmes MH (2001) DNA sequence quality trimming and vector removal. Bioinformatics 17: 1093–1104. 11751217 10.1093/bioinformatics/17.12.1093

[32] Niu BF, Fu LM, Sun SL, Li WZ (2010) Artificial and natural duplicates in pyrosequencing reads of metagenomic data. BMC Bioinformatics 11: 187. 20388221 10.1186/1471-2105-11-187 PMC2874554

[33] Phillippy AM, Schatz MC, Pop M (2008) Genome assembly forensics: finding the elusive mis-assembly. Genome Biol 9: R55. 18341692 10.1186/gb-2008-9-3-r55 PMC2397507

[34] Lowe TM, Eddy SR (1997) tRNAscan-SE: A program for improved detection of transfer RNA genes in genomic sequence. Nucleic Acids Res 25: 955–964. 9023104 10.1093/nar/25.5.955 PMC146525

[35] Thompson JD, Gibson TJ, Plewniak F, Jeanmougin F, Higgins DG (1997) The CLUSTAL_X windows interface: flexible strategies for multiple sequence alignment aided by quality analysis tools. Nucleic Acids Res 25: 4876–4882. 9396791 10.1093/nar/25.24.4876 PMC147148

[36] Kimura M (1980) A simple method for estimating evolutionary rates of base substitutions through comparative studies of nucleotide sequences. J Mol Evol 16: 111–120. 7463489 10.1007/BF01731581

[37] Tamura K, Peterson D, Peterson N, Stecher G, Nei M, et al (2011) MEGA5: molecular evolutionary genetics analysis using maximum likelihood, evolutionary distance, and maximum parsimony methods. Mol Biol Evol 28: 2731–2739. 21546353 10.1093/molbev/msr121 PMC3203626

[38] Darling ACE, Mau B, Blatter FR, Perna NT (2004) Mauve: multiple alignment of conserved genomic sequence with rearrangements. Genome Res 14: 1394–1403. 15231754 10.1101/gr.2289704 PMC442156

[39] Guindon S, Gascuel O (2003) A simple, fast and accurate algotithm to estimate large phylogenies by maximum likelihood. Syst Biol 52: 696–704. 14530136 10.1080/10635150390235520

[40] Adachi J, Waddell PJ, Martin W, Hasegawa M (2000) Plastid genome phylogeny and a model of amino acid substitution for proteins encoded by chloroplast DNA. J Mol Evol 50: 348–358. 10795826 10.1007/s002399910038

[41] Jones D, Taylor W, Thornton J (1992) The rapid generation of mutation data matrices from protein sequences. Comput Appl Biosci 8: 275–282. 1633570 10.1093/bioinformatics/8.3.275

[42] Ronquist F, Teslenko M, van der Mark P, Ayres DL, Darling A, et al (2012) MrBayes 3.2: efficient Bayesian phylogenetic inference and model choice across a large model space. Syst Biol 61: 539–542. 22357727 10.1093/sysbio/sys029 PMC3329765

[43] Puerta MVS, Bachvaroff TR, Delwiche CF (2005) The complete plastid genome sequence of the haptophyte *Emiliania huxleyi*: a comparison to other plastid genomes. DNA Res 12: 151–156. 16303746 10.1093/dnares/12.2.151

[44] Moon DA, Goff LJ (1997) Molecular characterization of two large DNA plasmids in the red alga *Porphyra pulchra* . Curr Genet 32: 132–138. 9294261 10.1007/s002940050257

[45] Douglas SE, Penny SL (1999) The plastid genome of the cryptophyte alga, *Guillardia theta*: complete sequence and conserved synteny groups confirm its common ancestry with red algae. J Mol Evol 48: 236–244. 9929392 10.1007/pl00006462

[46] Khan H, Parks N, Kozera C, Curtis BA, Parsons BJ, et al (2007) Plastid genome sequence of the cryptophyte alga *Rhodomonas salina* CCMP1319: lateral transfer of putative DNA replication machinery and a test of chromist plastid phylogeny. Mol Biol Evol 24: 1832–1842. 17522086 10.1093/molbev/msm101

[47] Grasser KD, Ritt C, Krieg M, Fernandez S, Alonso JC, et al (1997) The recombinant product of the *Cryptomonas* plastid gene *hlpA* is an architectural HU-like protein that promotes the assembly of complex nucleoprotein structures. Eur J Biochem 249: 70–76. 9363755 10.1111/j.1432-1033.1997.00070.x

[48] Simpson CL, Stern DB (2002) The treasure trove of algal chloroplast genomes. Surprises in architecture and gene content, and their functional implications. Plant Physiol 129: 957–966. 12114552 10.1104/pp.010908 PMC1540241

[49] Wang SL, Liu X-Q (1991) The plastid genome of *Cryptomonas* Φ encodes an hsp70-like protein, a histone-like protein, and an acyl carrier protein. Proc Natl Acad Sci U S A 88: 10783–10787. 1961745 10.1073/pnas.88.23.10783 PMC53015

[50] Gaikwad A, Hop DV, Mukherjee SK (2002) A 70-kDa chloroplast DNA polymerase from pea (*Pisum sativum*) that shows high processivity and displays moderate fidelity. Mol Genet Genomics 267: 45–56. 11919714 10.1007/s00438-001-0631-8

[51] Wattier RA, Prodöhl PA, Maggs CA (2000) DNA isolation protocol for red seaweed (Rhodophyta). Plant Mol Biol Rep 18: 275–281.

[52] Huang J, Gogarten JP (2008) Concerted gene recruitment in early plant evolution. Genome Biol 9: R109. 18611267 10.1186/gb-2008-9-7-r109 PMC2530860

[53] Rice DW, Palmer JD (2006) An exceptional horizontal gene transfer in plastids: gene replacement by a distant bacterial paralog and evidence that haptophyte and cryptophyte plastids are sisters. BMC Biol 4: 31. 16956407 10.1186/1741-7007-4-31 PMC1570145

[54] Yoon HS, Muller KM, Sheath RG, Ott FD, Bhattacharya D (2006) Defining the major lineages of red algae (Rhodophyta). J Phycol 42: 482–492.

[55] Broom J, Jones W, Hill D, Knight G, Nelson W (1999) Species recognition in New Zealand *Porphyra* using 18S rDNA sequencing. J Appl Phycol 11: 421–428.

[56] Sutherland JE, Lindstrom SC, Nelson WA, Brodie J, Lynch MDJ, et al (2011) A new look at an ancient order: generic revision of the Bangiales (Rhodophyta). J Phycol 47: 1131–1151. 27020195 10.1111/j.1529-8817.2011.01052.x

[57] Oliveira MC, Bhattacharya D (2000) Phylogeny of the Bangiophycidae (Rhodophyta) and the secondary endosymbiotic origin of algal plastids. Am J Bot 87: 482–492. 10766719

[58] Müller KM, Oliveira MC, Sheath RG, Bhattacharya D (2001) Ribosomal DNA phylogeny of the Bangiophycidae (Rhodophyta) and the origin of secondary plastids. Am J Bot 88: 1390–1400. 21669670

[59] Saunders GW, Hommersand MH (2004) Assessing red algal supraordinal diversity and taxonomy in the context of contemporary systematic data. Am J Bot 91: 1494–1507. 21652305 10.3732/ajb.91.10.1494

[60] Le Corguillé G, Pearson G, Valente M, Viegas C, Gschloessl B, et al (2009) Plastid genomes of two brown algae, *Ectocarpus siliculosus* and *Fucus vesiculosus*: further insights on the evolution of red-algal derived plastids. BMC Evol Biol 9: 253. 19835607 10.1186/1471-2148-9-253 PMC2765969

[61] Pinto G, Albertano P, Ciniglia C, Cozzolino S, Pollio A, et al (2003) Comparative approaches to the taxonomy of the genus *Galdieria Merola* (Cyanidiales, Rhodophyta). Cryptogam., Algol. 24: 13–32.

[62] Yoon HS, Hackett JD, Ciniglia C, Pinto G, Bhattacharya D (2004) A molecular timeline for the origin of photosynthetic eukaryotes. Mol Biol Evol 21: 809–818. 14963099 10.1093/molbev/msh075

[63] Sanchez-Puerta MV, Bachvaroff TR, Delwiche CF (2007) Sorting wheat from chaff in multi-gene analyses of chlorophyll c-containing plastids. Mol Phylogenet Evol 44: 885–897. 17449283 10.1016/j.ympev.2007.03.003

