# Supplementary material for: Complete Sequence and Analysis of Plastid Genomes of Two Economically Important Red Algae: Pyropia haitanensis and Pyropia yezoensis
Source: PLoS One. 2013 May 29;8(5):e65902. doi: 10.1371/journal.pone.0065902 (PMC3667073; doi:10.1371/journal.pone.0065902)
Supplement: Table S1 — Primers used for gap filling and assembly validation. (DOC) [file pone.0065902.s001.doc]

**Table S1.** Primers used for gap filling and assembly validation.

| 1F | CAATTCACCAGCCATAACTTCAT | 1R | CTGTCGATCCAGAGTTAATTGGT |
| --- | --- | --- | --- |
| 2F | AATGTCGGAGCTGTTGTCATATT | 2R | TTAACCTTAAGTTCAAGACCTGG |
| 3F | TTAATCTCTTGGCGTGGTTATTG | 3R | ATGCTATTGCAGTAGGACCAATC |
| 4F | ATTAATTGAGCACAATGCTCTTG | 4R | ACTCGTGCAATCTATTCTGGAAC |
| 5F | GCTACGGAATGGATTCTCTAAT | 5R | ATAGAACCGCATCCGTATTACC |
| 6F | TTAAGTGATGGACATAGCTGGAC | 6R | GACGTGCTTCCTCTAAAGACAAT |
| 7F | AAGATGCAAGAGTCGAAGTTGAA | 7R | GCCGAACTCTTCTAATGTAGGTG |
| 8F | TGCCTCCATAATTATTACTTCATT | 8R | AATCTGATAAGTTATGCCAACCG |
| 9F | ATTCTTCTTATGCTGGCCTCATC | 9R | ATTGAACTTGCATGTGTTAGGCT |
| 10F | GCACCTATTTCTAGTCCAGTAATCCTGTC | 10R | GAAGGTAATGGGCAACTTGTTCAATC |
| 11F | GGCAAGATGCTAATAGATGGAGC | 11R | CTCTTCTTGTAGCACACCGATCT |
| 12F | AGATCGGTGTGCTACAAGAAGAG | 12R | GCTTGGTCTGTAGTTATCGCATC |
| 13F | TTAGTTGTTGGTGGTACTGGTCC | 13R | AACTAACATCGCAGTAAGCAAGC |
| 14F | GCAAGGACAAGCTCTAACACATT | 14R | AGTTGAAGGTGTAGGAGATCACG |
| 15F | TTCTAATCCAAGTCTGGCACAAT | 15R | TCTTGGACAATCGATACAATCTG |
| 16F | CGCCAATGTGATACTTGTATTCG | 16R | GCTGCTCTCTGTACCTACCATTG |
| 17F | TACTAGATGTTGCGTGTATTGACCC | 17R | TGATCTGCGACAGCTTGTCCTACAT |
| 18F | AACCCATCCTACCGAATCACA | 18R | CTCGACCTACCCAACCAATCC |
| 19F | AAGACATCTAGCTCCTTCTTCTAC | 19R | TACGAGTTCGATTCTCGTCAT |
| 20F | TTATGGGAGGAAGTATGGGATC | 20R | TTTCTGGGTATGTGAAGGTGA |
| 21F | ACAGCATTTATCCTTGGGACG | 21R | AGACCGCGAGCAATTTACGA |
| 22F | AAAGTAAACTTCCCGCCTCA | 22R | GCTGCATAATTTGTTGCCGTA |
| 23F | TTATTGTTCCGACCTTCTGCTGT | 23R | TATGGCCTGTAGTTGGTATCTGG |
| 24F | AACGACCGAAGTAGCCATGAGC | 24R | TCTACGATTCCTGGGCAAACAG |
| 25F | AAGGGCATGTAACTCAGTGGAT | 25R | TAGAATCGGGTACAGCTACCAAC |
| 26F | ATCAGCGAAATGACTTGGTTGT | 26R | TACGATGGTAGTTCAGGGAGAC |
| 27F | TCTATGGCTTCAGATTCTCCATTCGG | 27R | TCCATTTTCCTTTGCTTCGGTCA |
| 28F | ATCTGTAGGACGAGATGTAGACGC | 28R | GAGATTGGCCCATGATGGACTAC |
| 29F | CTTCTAACTTTTCCTGCCGTTA | 29R | TTCGCTAGTTCAATCCAAGCAC |
| 30F | GCCTGTCTAACACTGTAACGAGG | 30R | CCTTAACAACAGTCATCCCTCC |
| 31F | CCAGGCATAATTGAAACGACAT | 31R | TTGAGATCGTGGCATAAGTGG |
| 32F | TACTAGGACGTATGCCATCTGC | 32R | CTTATGGAATGTTAGCTGGAGA |
| 33F | TTGGTGGAAATAGAGGTAGGG | 33R | TTAAGAGCGGCAACAGATGAG |
| 34F | TAATTGTCTACCAACTCCTCCCG | 34R | GATGTTTCGCCGCTGCGTATA |
| 35F | CCCAGACTCTTCATAGAATACCG | 35R | TCTATTGCGATGGTGGTTTGG |
| 36F | TGGGCGAAATGTTAATCCTGT | 36R | CGAACTATCGCAGAAAGAGGAG |
| 37F | GGCGACCTAAATCCATCACTAC | 37R | AAGCCACCACCTACATCTCCAT |
| 38F | TCCACTAGCACCAGAGGATGAC | 38R | CCGAACAGTTACTTGCCTTTGA |
| 39F | TAGACTTGTGAGCGAGCTGTT | 39R | GTTGCGGATAATAGTGGTGCT |
| 40F | GCACCACTATTATCCGCAACA | 40R | CAGAGGCAGAATGAAAGGTTC |
| 41F | CTCCCTTAAACAGTGATAGTGC | 41R | CCAGTAATACTCCATCCTCCC |
| 42F | TTGGGGAACAACTCCTATCA | 42R | AAGCTAAGCCTCCAACTAACA |
| 43F | CTTGGGTAGAGGAATGGGAGGAG | 43R | GGAAGAATAGCTCCGCCTGGAT |
| 44F | TGGCAGAACTTGTAGTTGGATG | 44R | TTAGTACAGAAGCAGGAGGAGTTT |
| 45F | TAAACGTCCCTGCTTCTTGTCCACA | 45R | AAGAAGTCGTGCCAGATGTTCGCT |
| 46F | CAAATGATGTCGCTGGTGATG | 46R | TGGAAATGGTACAAGTCCACTTAC |
| 47F | TTCTTCCCAAGCATCATTCCT | 47R | GAACTGCTTGCTCAGAACTAGG |
| 48F | CCTTTAGGAGAGGCATAACAGT | 48R | CATTGTGGAGATAAGCGGACT |
| 49F | GTGTAGGCGTTAGAGTATTGAG | 49R | GCTTAGTATCCGCTTAGTGTC |
| 50F | TACTGTATGAGGCGAAGAAAG | 50R | CGTTCAAAGAATACTGGGTAG |
| 51F | ACGGTATCATTGAGAAGTCCTG | 51R | TGAGCGTCCGAGTTTATCTTG |
| 52F | CCCATTATGCGATACAAAACG | 52R | GCTCGGTTAGCAACTTGAACTG |
| 53F | TAAGTAATGCGGTCCAGTTAG | 53R | GTTAGAAAAGCCACACATAGG |
| 54F | GGCAGTTCCAATATGTAGCAGA | 54R | AGATAGGAGGTCGGCATGAGTG |
| 55F | TCAGCAATGGTAGACACGAGAG | 55R | TTTGTTAGTTGTCAGTGGGGAT |
| 56F | GGCGATAGTGCTGTAGTAGATGT | 56R | CAAGGATGGAAAAGATGCGAT |
| 57F | CCTCGGGACATAAGTCAGTAGT | 57R | ACACTGTTTGTCACTCCATCTC |
| 58F | TTGTTGATTGTCAGAGCAGTGT | 58R | GCAAAAACGCTGTCAGGATACT |
| 59F | TTAATATAGAGCGTCCACATGGTGT | 59R | AAGCTAATTGACCTTCAATAATAGC |
| 60F | ATTGCAGGAACTGGACATAATCA | 60R | AAGCAGATCAGCTAACTCAACAA |
| 61F | AAGTATTACGCAACAAGTTCTCAG | 61R | ATAATCGACTGACCTGGATCAAC |
| 62F | CTTCTGCTGTAGGTGTCGATGAG | 62R | TTCTCGTGCACTAGTGGAATTAT |
| 63F | TGAACAGTTATCTGCATCACCAG | 63R | TCTTGATTATCCAATCGTTCTTG |
| 64F | GGCCGAGTGATATCTTGATAGTG | 64R | ACACCTGGTTGAGGAGTGATTCT |
| 65F | ACAATTAGCTGTCCAACAAGG | 65R | GAAGGCAGCGGACTCATAATC |
| 66F | TGGACTAGATATTGATGCATTACG | 66R | TATTCCAGTTGAAGAACAGACTGC |
| 67F | GCAATTACTAACCAAGAGACAATG | 67R | TCTAATCGTACCGCTATTGAAGG |
| 68F | GCTTGGCATAATAATTGGTTGAC | 68R | AACTACATCAATGTGGATTGCCA |
| 69F | TTGCTATTCTTGGTCTTGATGAAC | 69R | CTGCGCCATTAACCAGAATAGTT |
| 70F | TACTGTACTGCTATTGGCGGATT | 70R | TGATGAGCAGTATCACTTAGCCA |
| 71F | TAGTAGATGCTCTCGACCAAGTT | 71R | GTCATGAATCCTGTAGACCATCC |
| 72F | TCAGTTAATTGCAAGTCTCAATCC | 72R | TCGTATAATGCCATAGATCCTGC |
| 73F | ATTGGCCGGATTAATCACAGTT | 73R | GAATAAGAATCTGCAGCATGAGC |
| 74F | AATCCAAGCTATGGAGCTAGACC | 74R | CTACTCACAACACAAGGATGGAA |
| 75F | GGTTGTAGCTCAGAAGGATAGAGC | 75R | GAATAGCTCTGTTATACCATGAAGA |
| 76F | CCTAGTTCTGAGCAAGCAGTTCT | 76R | ATCAAGAACGATATTCAACCGTG |
| 77F | TAACTCACCGATCATGATTACCA | 77R | TCCAACTGTTATGCCTCTCCTAA |
| 78F | AAGTGGCTGATGTCATACTTCCTAG | 78R | CACGTCCTAAAGCTGCTATACGC |
| 79F | TACCTCGTAGCCATCTACACGTT | 79R | CGAGCCGATGTATTAGTATCGAA |
| 80F | CTCAAGCAGGTGTTGAATATGCT | 80R | AATTGCTGTAGCTCCAATGAGTC |
| 81F | ACAATCTTCAGTTCTTGGTGCAG | 81R | GTTGTTCCACCTTCAGAAGCTAA |
| 82F | AAGCTGGTACGTTGAAGCACTAC | 82R | ATCCATTGCATAATATCGACCAC |
| 83F | TATTCTTCCTGTTCCAGCCATAA | 83R | CATGGATTAGCAATTCCAACTGT |
| 84F | TGTTGACTTACCTGTTCCACGAT | 84R | CTACGCATTTCACCGCTACACT |
| 85F | TGGACAGAAAGACCCTATGAAG | 85R | AACCTCCACATACAACATCACC |
| 86F | CAGCAGGTTCTAGGAAAAGATCG | 86R | CTAACGCTAAAGGGATTGACGC |
| 87F | TACTAGCCCGTATCGTAGGTGC | 87R | AGGAGAAATAGCAGCCGCTCT |
| 88F | GTTAACATTTTCGAGTGCTGCTCT | 88R | ATATTGTTAGAGTTCGGGGTGG |
| 89F | TAATAAGCTCGCCAGTCTACTG | 89R | AATAACCATAGCACCCCATGTC |
| 90F | GTAAACCCAGAAGAGAGCAACC | 90R | GTCGTGCAACTTAAACATAGCGT |
| 91F | AGACCTCCAAAGCCAAGAACAG | 91R | AACAGCATGGGACATTCACTTC |
| 92F | TCGGAAATCAGTCGTAGAGTGT | 92R | TATCCAGTAGCAGCAATCCCTT |
| 93F | AGCTAATCTTCCAAGTAATCCC | 93R | CGCTAATAACCAAAACCTACTC |
| 94F | TAACAGCCGATTGCTCTACCAC | 94R | CAACTCTGGAAAATGCGTCAAG |
| 95F | CCTTTAGGAGAGGCATAACAGT | 95R | CATTGTGGAGATAAGCGGACTC |

Primer pairs 1-17 and 59-85 were used to finish gaps in the assembly of *P*. *haitanensis* and *P*. *yezoensis*, respectively. Primer pairs 18-58 and 86-95 were used to verify the assembly of *P*. *haitanensis* and *P*. *yezoensis*, respectively.
