# Supplementary material for: Complete Sequence and Analysis of Plastid Genomes of Two Economically Important Red Algae: Pyropia haitanensis and Pyropia yezoensis
Source: PLoS One. 2013 May 29;8(5):e65902. doi: 10.1371/journal.pone.0065902 (PMC3667073; doi:10.1371/journal.pone.0065902)
Supplement: Table S2 — Plastid and cyanobacterial genomes used in the gene content comparisons and the phylogenetic studies. (DOC) [file pone.0065902.s002.doc]

**Table S2.** Plastid and cyanobacterial genomes used in the gene content comparisons and the phylogenetic studies.

| Species | Accession Number | Date |
| --- | --- | --- |
| *Synechocystis* sp. PCC 6803 | NC_000911 | 01/19/2012 |
| *Prochlorococcus marinus* | NC_009976 | 01/19/2012 |
| *Cyanidioschyzon merolae* | NC_004799 | 04/15/2009 |
| *Cyanidium caldarium* | NC_001840 | 05/06/2009 |
| *Gracilaria tenuistitipata* | NC_006137 | 04/15/2009 |
| *Porphyra purpurea* | NC_000925 | 03/26/2010 |
| *Pyropia yezoensis* | KC517072 | this study |
| *Cyanophora paradoxa* | NC_001675 | 04/15/2009 |
| *Guillardia theta* | NC_000926 | 05/06/2009 |
| *Rhodomonas salina* | NC_009573 | 03/26/2010 |
| *Emiliania huxleyi* | NC_007288 | 05/03/2010 |
| *Thalassiosira pseudonana* | NC_008589 | 03/26/2010 |
| *Odontella sinensis* | NC_001713 | 03/26/2010 |
| *Heterosigma akashiwo* | NC_010772 | 05/14/2009 |
| *Fucus vesiculosus* | NC_016735 | 02/17/2012 |
| *Ectocarpus siliculosus* | NC_013498 | 11/21/2009 |
| *Chlamydomonas reinhardtii* | NC_005353 | 01/23/2004 |
| *Nephroselmis olivacea* | NC_000927 | 04/15/2009 |
| *Chlorella vulgaris* | NC_001865 | 04/15/2009 |
| *Mesostigma viride* | NC_002186 | 04/15/2009 |
| *Arabidopsis thaliana* | NC_000932 | 03/26/2010 |
| *Saccharina japonica* | NC_018523 | 09/04/2012 |
| *Vaucheria litorea* | NC_011600 | 03/26/2010 |
| *Phaeodactylum tricornutum* | NC_008588 | 03/26/2010 |
| *Aureoumbra lagunensis** | NC_012903 | 07/02/2010 |
| *Bryopsis hypnoides** | NC_013359 | 08/01/2011 |
| *Pyramimonas parkeae** | NC_012099 | 09/29/2011 |
| *Oryza rufipogon** | NC_017835 | 05/09/2012 |
| *Phaeocystis Antarctica** | NC_016703 | 01/27/2012 |
| *Pyropia haitanensis* | KC464603 | this study |

(*) Species only used in comparisons of gene contents between plastid genomes**.**
