# Supplementary material for: Complete Sequence and Analysis of Plastid Genomes of Two Economically Important Red Algae: Pyropia haitanensis and Pyropia yezoensis
Source: PLoS One. 2013 May 29;8(5):e65902. doi: 10.1371/journal.pone.0065902 (PMC3667073; doi:10.1371/journal.pone.0065902)
Supplement: Table S3 — Gene content comparisons between plastid genomes. (DOC) [file pone.0065902.s003.doc]

**Table S3. Gene content comparisons between plastid genomes**.

| Genes | Species | | | | | | | | | | | | | | | | | | | | |
| --- | --- | --- | --- | --- | --- | --- | --- | --- | --- | --- | --- | --- | --- | --- | --- | --- | --- | --- | --- | --- | --- |
|  | No | Rhodophytes | | | | | | Heterokonts | | | | Cryptophytes | | Haptophytes | | Chlorophytes | | | | Streptophytes | |
|  |  | Cmer | Ccal | Ppur | Pyez | Phai | Gten | Esil | Osin | Vlit | Alag | Rsal | Gthe | Ehux | Pant | Crei | Bhyp | Cvul | Ppar | Atha | Oruf |
| Genes shared by all plastid genomes* | 40 | + | + | + | + | + | + | + | + | **+** | **+** | + | + | + | + | + | + | + | + | + | + |
| Genes shared by many plastid genomes of red and green lineages | 30 |  |  |  |  |  |  |  |  |  |  |  |  |  |  |  |  |  |  |  |  |
| *rpoC1*, *rpoC2* | 2 | - | + | + | + | + | + | + | + | **+** | **+** | + | + | + | + | + | + | + | + | + | + |
| *rps9*, *tufA*, *ycf12* | 3 | + | + | + | + | + | + | + | + | **+** | **+** | + | + | + | + | + | + | + | + | - | - |
| *petN*, *psaI*, *rpl22*, *rpl5*, *rps16*, *petG*, *psaJ*, *psbT*, *petD, petB*, *rpl2*, *rpl12, rps12*, *rps19*, *rpl32, ycf3*, *chlI*, *rpl19*, *psaM*, *rbcR* | 20 | + | + | + | + | + | + | + | + | + | +/- | + | + | +/- | +/- | - | +/- | +/- | +/- | +/- | +/- |
| *psbZ* | 1 | + | - | + | + | + | - | - | - | **+** | **-** | + | + | + | + | + | + | + | + | + | - |
| *cemA* | 1 | + | + | + | + | + | + | - | - | **-** | **-** | + | + | - | - | + | + | + | - | + | + |
| *chlN* | 1 | - | + | + | + | + | - | + | - | **+** | **+** | - | - | - | - | + | + | + | + | - | - |
| *chlB*, *chlL* | 2 | - | - | + | + | + | - | + | - | + | + | - | - | - | +/- | + | + | + | + | - | - |
| Genes mainly encoded by plastid genomes of the red lineage | 85 |  |  |  |  |  |  |  |  |  |  |  |  |  |  |  |  |  |  |  |  |
| *ycf16*, *17*, *19*, *20*, 23, 24, 59, 65, *rps6*, *rpl28*, *cbbX*, *ccs1*, *tatC*, *rpl33*, *secA* | 15 | + | + | + | + | + | + | +/- | +/- | +/- | **+/-** | **+/-** | **+/-** | **+/-** | **+/-** | - | - | **-** | **-** | +/- | - |
| *ycf21*, *35*, *36*, *46* | 4 | - | - | + | + | + | + | +/- | +/- | +/- | **+/-** | +/- | +/- | +/- | +/- | - | - | **-** | **-** | - | - |
| *atpD*, *atpG*, *dnaK*, *groEL*, *psaF*, *psbV*, *psaL*, *psaD*, *rbcS*, *rpl3*, *rpl6*, *rpl21*, *rpl27*, *rpl31*, *rpl34*, *rps5*, *rps10*, *rps13*, *rps17*, *clpC*, *secY*, *ycf39* | 22 | + | + | + | + | + | + | + | + | **+** | **+** | + | + | + | + | - | - | **-** | **-** | - | - |
| *ycf7*, *31* | 2 | - | - | + | + | + | - | - | +/- | +/- | - | - | +/- | +/- | - | - | - | - | - | - | - |
| *ycf22*, *29*,*38* | 3 | + | - | + | + | + | + | - | - | **-** | **-** | +/- | +/- | +/- | - | - | - | **-** | **-** | - | - |
| *ycf18*, *26* | 2 | - | + | + | + | + | - | - | - | **-** | **-** | +/- | - | +/- | - | - | - | **-** | **-** | - | - |
| *ycf32*, *37*, *61* | 3 | - | + | + | + | + | + | +/- | - | +/- | **+/-** | + | **+/-** | + | - | - | - | **-** | **-** | - | - |
| *ftsH* | 1 | + | + | + | + | + | + | + | + | + | **-** | + | + | - | - | - | - | + | + | - | - |
| *dnaB*, *rpl4*, *rpl13*, *rpl24*, *rpl29*, *tsf* | 6 | + | + | + | + | + | + | + | + | + | **+/-** | + | + | - | - | - | - | - | - | - | - |
| *acpP* | 1 | + | + | + | + | + | + | - | + | + | - | + | + | - | - | - | - | - | - | - | + |
| *petF*, *psaE*, *rpl1*, *rpl11*, *rpl18*, *rpl35*, *psbW* | 7 | + | + | + | + | + | + | + | + | **+/-** | **+/-** | + | + | - | - | - | - | - | - | - | - |
| *psbX*, *ycf33*, *rps20* | 3 | + | - | + | + | + | + | + | + | **+** | **+/-** | + | + | - | - | - | - | - | - |  |  |
| *ftrB*, *ilvB*, *ilvH* | 3 | + | + | + | + | + | + | + | - | **+/-** | **+/-** | +/- | +/- | - | - | - | - | - | - | - | - |
| *infC*, *carA*, *psaK* | 3 | + | + | + | + | + | + | - | - | **-** | **-** | +/- | +/- | - | - | - | - | - | - | - | - |
| *pbsA*, *cpeB*, *rne* | 3 | - | - | + | + | + | + | - | - | **-** | **-** | + | + | - | - | - | - | - | - | - | - |
| *infB* | 1 | + | - | + | + | + | + | - | - | **-** | **-** | + | + | - | - | - | - | - | - | - | - |
| *syfB*, *ycf34*, *rpl9* | 3 | - | - | + | + | + | + | + | - | **+/-** | **-** | - | - | - | - | - | - | - | - | - | - |
| *thiG*, *petJ* | 2 | + | + | + | + | + | + | + | +/- | **+** | **-** | - | - | +/- | - | - | - | - | - | - | - |
| *rps1* | 1 | + | - | + | + | + | + | + | - | **+** | **-** | - | - | - | - | - | - | - | - |  |  |
| Genes only present in red algal plastid genomes (except for *accD*) | 26 |  |  |  |  |  |  |  |  |  |  |  |  |  |  |  |  |  |  |  |  |
| *trxA* | 1 | - | + | + | + | + | + | - | - | **-** | **-** | - | - | - | - | - | - | - | - | - | - |
| *apcA*, *apcB*, *apcD*, *apcE*, *apcF*, *cpcA*, *cpcB*, *cpcG*, *preA*, *trpG* | 10 | + | + | + | + | + | + | - | - | **-** | **-** | - | - | - | - | - | - | - | - | - | - |
| *accA*, *accB*, *trpA*, *odpA*, *odpB*, *argB*, *gltB*, | 7 | + | + | + | + | + | + | - | - | **-** | **-** | - | - | - | - | - | - | - | - | - | - |
| *accD* | 1 | + | + | + | + | + | + | - | - | **-** | **-** | - | - | - | - | - | + | - | - | - | + |
| *cpeA*, *fabH*, *syh,* *pgmA* | 4 | - | - | + | + | + | + | - | - | **-** | **-** | - | - | - | - | - | - | - | - | - | - |
| *ycf28* | 2 | + | - | + | + | + | - | - | - | **-** | **-** | - | - | - | - | - | - | **-** | **-** | - | - |
| *glnB* | 1 | - | + | + | + | + | - | - | - | **-** | **-** | - | - | - | - | - | - | - | - | - | - |
| ORFs in the Bangiales | 31 |  |  |  |  |  |  |  |  |  |  |  |  |  |  |  |  |  |  |  |  |
| *ORF174*, *238*, *320*, *108*, *263*, *121*, *114*, *450*, *58*, *62*, *621*, *149*, *111*, *287*, *199*, *71*, *565*, *68*, *382*, *75*, *148*, *198*, *27*, *203*, *240*, *327*, *107* | 27 | - | - | + | + | + | - | - | - | **-** | **-** | - | **-** | - | **-** | - | **-** | **-** | **-** | - | - |
| *ORF32*, *ORF36* | 2 | - | - | - | + | + | - | - | - | **-** | **-** | - | **-** | - | **-** | - | **-** | **-** | **-** | - | - |
| *ORF33*, *ORF35* | 2 | - | - | - | + | - | - | - | - | **-** | **-** | - | **-** | - | **-** | - | **-** | **-** | **-** | - | - |

*P*. *haitanensis* and *P*. *yezoensis* plastid genomes were compared with those of other rhodophytes, heterokonts, haptophytes, cryptophytes, chlorophytes, streptophytes. Presence (+), absence (-) or presence of only a subset (+/-) of the genes listed in the first column is indicated. Species abbreviations: *Cyanidioschyzon merolae* (Cmer), *Cyanidium caldarium* (Ccal), *Porphyra purpurea* (Ppur), *Pyropia yezoensis* (Pyez), *Pyropia haitanensis* (Phai), *Gracilaria tenuistitipata* (Gten), *Ectocarpus siliculosus* (Esil), *Odontella sinensis* (Osin), *Vaucheria litorea* (Vlit), *Aureoumbra lagunensis* (Alag), *Rhodomonas salina* (Rsal), *Guillardia theta* (Gthe), *Emiliania huxleyi* (Ehux), *Phaeocystis antarctica* (Pant), *Chlamydomonas reinhardtii* (Crei), *Bryopsis hypnoides* (Bhyp), *Chlorella vulgaris* (Cvul), *Pyramimonas parkeae* (Ppar), *Arabidopsis thaliana* (Atha), *Oryza rufipogon* (Oruf). (*) A common core set of 40 genes are shared by all compared plastid genomes: *atpA*, *B*, *E*, *F*, *H*, *I*, *petA*, *psaA*, *B*, *C*, *psbA*, *B*, *C*, *D*, *E*, *F*, *H*, *I*, *J*, *K*, *L*, *N*, *rpl14*, *16*, *20*, *23*, *36*, *rpoA*, *B*, *rps2*, *3*, *4*, *7*, *8*, *11*, *14*, *18*, *ycf4*, *ccsA*, *rbc*L.
